# Differences in Sole Carbon Source Utilization of the Dental Plaque Microbiota Between Caries-Free and Caries-Affected Children

**DOI:** 10.3389/fmicb.2020.00458

**Published:** 2020-03-20

**Authors:** Jing Tian, Weihua Shi, He Xu, Guiyan Wang, Xuesong He, Feng Chen, Man Qin

**Affiliations:** ^1^Department of Pediatric Dentistry, Peking University School and Hospital of Stomatology, Beijing, China; ^2^The Forsyth Institute, Cambridge, MA, United States; ^3^Central Laboratory, Peking University School and Hospital of Stomatology, Beijing, China; ^4^National Engineering Laboratory for Digital and Material Technology of Stomatology, Beijing Key Laboratory of Digital Stomatology, Peking University School and Hospital of Stomatology, Beijing, China

**Keywords:** childhood caries, oral microbiota, metabolism, sole carbon source utilization, biolog assay

## Abstract

Increasing lines of evidence indicate that while microbial profile might vary, community-level metabolic potential is often more stably correlated with healthy and diseased states. Here, we investigated the community-level metabolic diversity of dental plaque microbiota from caries-free (CF) and caries-affected (CA) children by measuring their sole carbon source utilization using a Biolog assay. The dietary habits of 32 CF and 31 CA children were recorded by a questionnaire. Supragingival plaque samples were collected and inoculated into Biolog AN Microplates to assess the metabolism of sole carbon sources by plaque bacteria. The results revealed significant differences in dietary habits between CF and CA children. Meanwhile, Biolog assay showed consistently higher, albeit not statistically significant, overall metabolic activity as measured by average well color development (AWCD) value in the plaque microbiota from CA group than CF group. Most importantly, the CA group had more than twice as many core-positive carbon sources (defined as being utilized by >90% of plaque microbiota from subjects within the group) as that of the CF group (31 vs. 14), including CA group-specific, cariogenic core-positive carbon sources such as sucrose, glucose and raffinose. Furthermore, CF and CA groups could be well distinguished by cluster and principle component analyses based on the types of sole carbon sources significantly differentially utilized by the two groups. Our results indicate that plaque communities associated with caries state are more metabolically versatile than those associated with healthy state, which could contribute to differential clinical caries states. Meanwhile, Biolog could be an effective tool in revealing the community-level physiological profiles of microbiota associated with different caries states.

## Introduction

The prevalence of childhood caries in China has increased in recent years, developing into an alarming situation. The Fourth National Oral Health Epidemiological Survey (Wang, 2018) (Du et al., 2018) reported that 50.8 and 71.9% of children developed caries by 3 and 5 years old, respectively. Meanwhile, 63.8% of 3–5-year-old children drank sweetened beverages more than once per day, while only 59.9% of them brushed their teeth every day. As the most common chronic disease in children, dental caries has a multifactorial nature; it usually occurs when sugar is metabolized by certain bacteria in dental plaque resulting in increased acid production and dental demineralization, and with time forming a dental cavity.

Studies have shown that (Gross et al., 2012; Fechney et al., 2019; Hurley et al., 2019) the overall microbial composition and structure, rather than any particular dominant species such as *Streptococcus mutans*, could better characterize the cariogenicity of oral biofilms. Furthermore, increasing lines of evidence suggest that while microbial profile might vary, community-level metabolic potential is often better correlated with healthy and diseased states. Our previous longitudinal study (Xu et al., 2018) showed that amino and nucleotide sugar metabolism, fructose and mannose metabolism, glycolysis/gluconeogenesis, and starch and sucrose metabolism exhibited higher levels of pathway relative abundance in dental plaque microbiome of caries-affected (CA) than caries-free (CF) children. However, previous studies have mainly focused on the microbial community structure and metabolic pathways of a limited number of carbon sources (Campbell and Zinner, 1970; Moynihan et al., 1998; Moye et al., 2014); while the overall community level metabolic potential of plaque microbiota is less well investigated.

Anderson et al. (2002) was the first to use a Biolog assay to analyze the metabolic diversity of dental plaque biofilms based on sole carbon source utilization. Other subsequent studies also focused on analyzing the metabolic activity of periodontitis and caries-associated microbiota using Biolog assays (Zhang et al., 2014; Zhao et al., 2017). The Biolog plate contains an array of carbon substrates, each in a separate well with tetrazole redox dye; when bacteria utilize carbon sources, the respiration effect causes dye change to purple and the substrate catabolism can be evaluated as an optical density (OD) increase than that in a control well with no substrate. Therefore, the utilization patterns of 95 sole carbon sources can be determined simultaneously using one Biolog assay.

Our recent study demonstrated that microbiome associated with CA group exhibited more abundant carbohydrate-related metabolic pathways than the CF group at the genomic level (Xu et al., 2018), we thus hypothesize that the microbiome associated with different caries states could display differential metabolic potential at the physiological level. The aims of this study are twofold: (1) to explore the physiological-level metabolic diversity of the plaque microbiota associated with CF and CA children; and (2) to investigate the differences in subjects’ dietary habits between these two groups. Our data clearly revealed the differences between CF and CA children, both in dental plaque microbial metabolic versatility and dietary habits, thus expanding our current knowledge regarding the different physiological profiles of microbial community associated with health and caries-state.

## Materials and Methods

### Ethics Statement

The Ethics Committee of the Peking University Health Science Center approved the design and protocol of this study (PKUSSIRB-201631129). Written informed consent was obtained from the participants’ parents or caregivers prior to enrollment.

### Participants

A total of 63 children were enrolled in this study. The inclusion criteria for all the participants were: healthy, 3- to 4-year old with primary dentition of more than 18 teeth. The exclusion criteria were: refusal to cooperate; systemic disease; having orthodontic appliance in the mouth; use of antibiotics or topical fluoride within 1 month. 32 CF children from five urban kindergartens in Beijing, China, who underwent routine oral examinations were recruited randomly as the CF group; 31 children with four or more decayed teeth (dt ≥ 4) and less than two filled teeth (df < 2), seeking caries treatment at Peking University School and Hospital of Stomatology were recruited randomly as the CA group. Caries states and the decayed teeth (dt) index, decayed, missing, filled teeth (dmft) index, decayed, missing, filled surfaces (dmfs) index were scored according to the modified World Health Organization (1997) caries diagnostic criteria. Children with white spots on their teeth were excluded from the CF group. The kappa value for intra-examiner agreement in the diagnosis of caries was 0.834.

### Sample Collection

Plaque samples were collected from 10:00–11:00 am. All participants were instructed not to brush their teeth in the morning and required to abstain from food and drink for 2 h prior sample collection after they ate the same breakfast in kindergarten, and to rinse their mouths before sample collection. The teeth were isolated with cotton rolls and gently air-dried, and supragingival plaque was collected from all sound smooth surfaces using a sterile dental excavator, and pooled into sterile 1.5 mL centrifuge tubes containing 1 mL phosphate-buffered saline (0.01 M, pH 7.2–7.4; Solarbio, Beijing, China). Samples were immediately shipped on ice to the microbiology laboratory at Peking University School and Hospital of Stomatology within 2 h. Questionnaires recording the participants’ oral behavioral habits were completed by the parents or caregivers.

### Biolog Assay

Supragingival plaque samples were re-suspended in 12 mL AN inoculating fluid (AN-IF, Catalog No. 72007; Biolog Inc., Hayward, CA, United States) and extensively vortexed for 1 min to ensure the breakup of the dental plaque and achieve even dispersion of plaque cells in the solution. Biolog AN plate instead of the Biolog GN plate was chosen according to a previous study, which indicated that the AN plate can better differentiate between CF and CA states (Zhao et al., 2017). The plaque suspensions were inoculated into a Biolog AN microplate (Catalog No. 1007; Biolog Inc.) at 100 μL per well. Each Biolog AN plate contained one control well with no carbon source (well A1) and 95 wells, each containing one sole carbon source (wells A2–H12) (Supplementary Table S1). Then plates were sealed with an anaerobic sachet bag and incubated at 37°C. Anaerobic condition was used for incubation since the reproducibility and rate of substrate utilization by plaque community under this condition were the most suitable attributes for characterization of the metabolic diversity of dental plaque (Anderson et al., 2002). During incubation, cellular respiration as a result of utilization of carbon source by the inoculated plaque microbiota causes the tetrazole redox dye in the wells to turn purple, and leads to change in OD at 590 nm. The OD_590_ in each well was recorded at 24, 48, 72, and 96 h during incubation using a Biolog MicroStation (Biolog OmniLog version 4.1). Safety training is required for anyone working in the central laboratory of Peking University Hospital of Stomatology and all standard biosecurity and institutional safety procedures must be followed under the supervision of the lab manager.

One technical limitation of Biolog assay, particularly when applied to plaque samples, as encountered in previous studies (Zhang et al., 2014; Zhao et al., 2017), is the requirement of sufficient amount of sample material which made the duplication or triplication of the experiment almost unlikely.

### Statistical Analyses

The demographic data, dmft scores, and dietary habits of the participants were analyzed using the SPSS software (version 24.0) with the Mann–Whitney *U*-test and chi-squared test. The overall metabolic activity of the oral microbiota on each Biolog AN plate was assessed by average well color development (AWCD) (Anderson et al., 2002), which was calculated using the following formula:

$$\text{AWCD}=\sum_{i=1}^{n}\frac{c_{i}}{n}$$

where *C* represents the corrected OD_590_ value in each well and *n* is the number of substrates (*n* = 95) for the 95 sole carbon source wells (*C*_*i*_ = [OD value of well *i*] – [OD value of control well A1]). To correct for the possible metabolism of carbon sources in the dental plaque, if the result was negative, the OD was listed as zero (Zhang et al., 2014). The Mann–Whitney *U*-test was used to compare the mean differences in AWCD between the CF and CA groups. The Biolog OmniLog software was used to identify the metabolic reactions, which were interpreted as positive or negative. Positive wells found in at least 90% of subjects in the CF or CA groups after 96 h incubation were defined as core-positive carbon sources. Although the plaque samples were extensively vortexed to achieve even dispersion, we further used transformed data (by dividing each corrected OD_590_ with the AWCD of the plate) to account for potential different inoculum density (Garland and Mills, 1991). Furthermore, transformed data is more likely to reflect differences among samples in the fraction, rather than absolute density of microorganisms in the inoculum that are able to utilize substrates as sole carbon sources, thus more useful than corrected OD_590_ value for classifying samples among and within microbial habitats (Garland and Mills, 1991). Comparison of the utilization of the 95 sole carbon sources between the CF and CA groups was conducted with the transformed data using Mann–Whitney *U*-test. Transformed data of the 10 significantly differentially consumed carbon sources between the CF and CA groups at 24 h after incubation was also used to perform heatmap cluster and PCAs using the R software and ggbiplot package. 24 h timepoint was chosen since it could best reflect the community-level utilization rate of specific carbon source. We used Mann–Whitney *U*-test instead of independent *t*-test in this study since the data lacked normality and homogeneity of variance.

## Results

### Demographics, dmft Scores, and Dietary Habits of the CF and CA Groups

The demographic data, dmft scores, and dietary habits of the participants are described in Table 1. In total, 32 CF children and 31 CA children were enrolled in this study. The age and sex of participants did not significantly differ between the CF and CA groups. The average dt, dmft, and dmfs score in the CA group was 11.26 ± 3.54, 11.29 ± 3.49, and 21.16 ± 10.33, respectively. Among the dietary habits, the age at which children were no longer fed at night was significantly delayed in the CA group compared to the CF group (*P* < 0.05); the CA group had more children who were fed while going to sleep and throughout the night (*P* < 0.05). The CA group also drank sweet beverages before or during sleep (*P* < 0.01) and ate sweet foods (*P* < 0.05) more frequently than the CF group.

**TABLE 1:** The demographic, dmft and oral behavior habits of the participants.

	**CF group**	**CA group**	***P*-value**
No. of participants	32	31	
Age (month)	43 ± 4.75	42.48 ± 5.97	0.778^*a*^
Gender (female/male)	13/19	15/16	0.535^*b*^
dt	0	11.26 ± 3.54	
dmft	0	11.29 ± 3.56	
dmfs	0	21.16 ± 10.33	
The age of stopping night feeding (month)	12.91 ± 9.34	18.13 ± 9.17	0.033^a*^
Feeding while go to sleep			0.000004^b**^
No	27	9	
Yes	4	22	
Feeding during night sleep			0.031^b*^
Less than 2 times	30	24	
More than 3 times	0	6	
Drinking sweet beverage before or during sleep			0.000149^b**^
Seldom/never	28	13	
More than 1–2 times per week	4	18	
The frequencies of eating sweet food			0.020^b*^
Seldom/never	13	5	
More than 3–4 times per week	17	26	

*^*a*^Mann-Whitney *U*-test; ^*b*^Chi-square test; ^∗^*P* < 0.05, ^∗∗^*P* < 0.01.*

### Metabolic Activity of the Dental Plaque Microbiota in the CF and CA Groups

**FIGURE 1:**
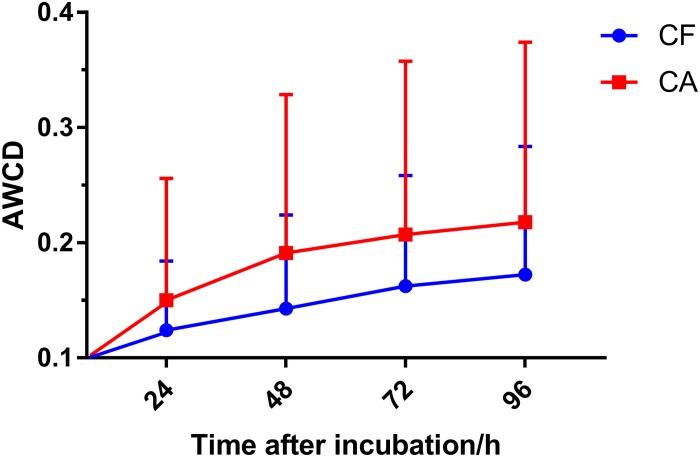
Average well color development (AWCD) value with incubation time in the CF and CA groups. Plaque microbiota samples were tested using Biolog AN plate, AWCD value was calculated at 24, 48, 72, and 96 h after incubation. Mean values ± SD are shown. Mann-Whitney *U*-test was used for statistical analysis (*P* > 0.05).

Biolog AN plate was used to test the overall metabolic activity of the plaque microbiota associated with the CA and CF group. Our data showed that although not statistically significant (*P* > 0.05, Mann–Whitney *U*-test), the AWCD values of the CA group were consistently higher than those of the CF group across all 4 timepoints measured (Figure 1). The original OD_590_ values of 63 children were attached in Supplementary Data.

### Core-Positive Carbon Sources Utilized by the Plaque Microbial Communities of the CF and CA Group

**FIGURE 2:**
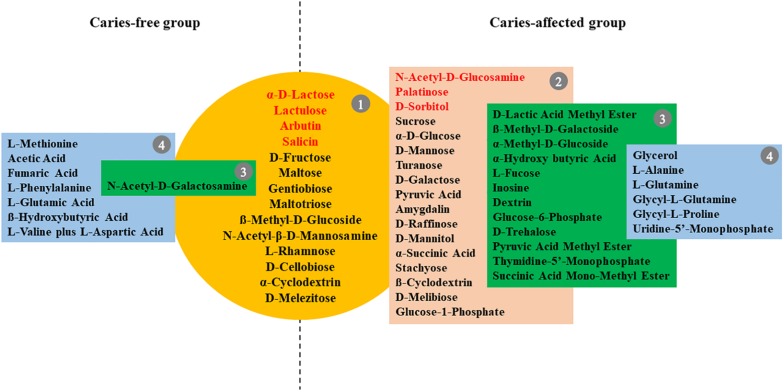
Core-positive carbon sources of CF and CA group. Metabolic reaction positive wells found in at least 90% of subjects in the CF or CA groups after 96 h incubation were defined as core-positive carbon sources. Group 1 (in yellow circle) are core-positive carbon sources shared by CF and CA; group 2 (in brown box) contains CA-specific core-positive carbon sources; seven carbon sources in group 1 and group 2 which were utilized by plaque microbiota of 100% subjects in the CA group were highlighted in red font. Carbon sources utilized by 80–90% of plaque microbiota from individuals in one group but by <80% in the other group are listed in group 3 (in green box); carbon sources utilized by 50–80% of plaque microbiota from individuals in one group but by <50% in the other group were listed in group 4 (in blue box).

We further took a closer look at the utilization pattern of sole carbon sources by plaque microbial communities of CF and CA group. The carbon source utilized by plaque communities from more than 90% of subjects in CA or CF group after 96 h incubation were defined as core-positive carbon sources of that particular group. Since it is possible that the relative abundance of particular bacterial species capable of using specific carbon source might be extremely low within certain plaque communities, it might take time for the metabolic reaction to be visually observed in the assay. Thus, the last time point (96 h) was chosen to best capture the overall carbon utilization. Our data showed that the CF and CA groups shared 14 core-positive carbon sources (category 1 in Figure 2). Furthermore, the CA group had additional 17 CA-specific core-positive carbon sources (category 2 in Figure 2), while no CF-specific core-positive carbon source could be found. Among the 31 core-positive carbon sources of the CA group, 7 were utilized by plaque microbiota from all the subjects in the CA group, including α-D-lactose, lactulose, arbutin, salicin, *N*-acetyl-D-glucosamine, palatinose, and D-sorbitol (highlighted in red font in Figure 2). For the non-core-positive carbon sources, those that were utilized by 80–90% of plaque microbiota from individuals in one group but by < 80% in the other group are listed in green boxes under their respective groups (category 3 in Figure 2). There are 12 and 1 carbon sources within this category for the CA and CF group, respectively. Furthermore, non-core-positive carbon sources utilized by 50–80% of plaque microbiota from individuals in one group but by <50% in the other group are listed in blue boxes under their respective groups (category 4 in Figure 2). Our data showed similar number of carbon sources within this category, with 6 and 7 for the CA and CF group, respectively. The original data of utilization percentages for the 95 sole carbon sources are presented in Supplementary Table S2.

### Differences in Utilization of Specific Carbon Sources Between the CF and CA Groups

**FIGURE 3:**
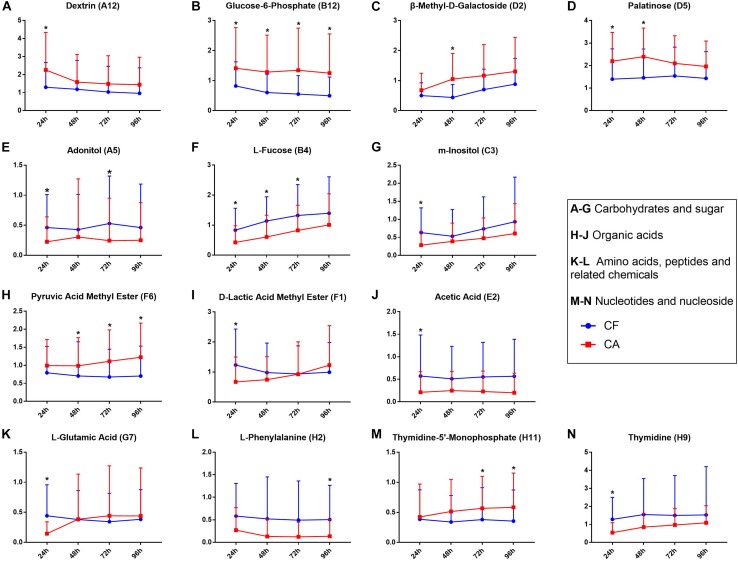
Carbon sources significantly differentially utilized between CF and CA groups. **(A–G)** were carbohydrates and sugar, **(H–J)** were organic acids, **(K,L)** were amino acids, peptides and related chemicals, **(M,N)** were nucleotides and nucleoside. Blue line represents CF group, red line represents CA group. All the *y*-axis represents transformed data, mean values ± SD were shown, Mann-Whitney *U*-test was used for statistical analysis, an asterisk (^∗^) indicates a significant difference between the two values (*P* < 0.05).

A detailed comparison revealed 14 sole carbon sources that displayed significant differences in utilization (*P* < 0.05) between the CF and CA groups at certain timepoints (24, 48, 72, and 96 h) following incubation (Supplementary Table S3). Among these carbon sources, seven were carbohydrates (Figures 3A–G), three were organic acids (Figures 3H–J), two were amino acids (Figures 3K,L), and two were nucleotide and nucleoside (Figures 3M,N). Among the carbohydrates, dextrin, glucose-6-phosphate, β-methyl-D-galactoside, and palatinose were overall utilized more by the CA group than by the CF group, whereas adonitol, L-fucose, and m-inositol were better catabolized by the CF group. Meanwhile, plaque communities from CA group were more capable of using pyruvic acid methyl ester and thymidine-5′-monophosphate (Figures 3H,M); CF group plaque microbiota displayed a faster initial rate in utilizing D-lactic acid methyl ester and L-glutamic acid as being reflected by the significantly higher value at 24 h compared to CA group, while reaching value similar to CA group at 96 h (Figures 3I,K).

### Correlation Between Microbial Carbon Source Utilization Patterns and Caries States

**FIGURE 4:**
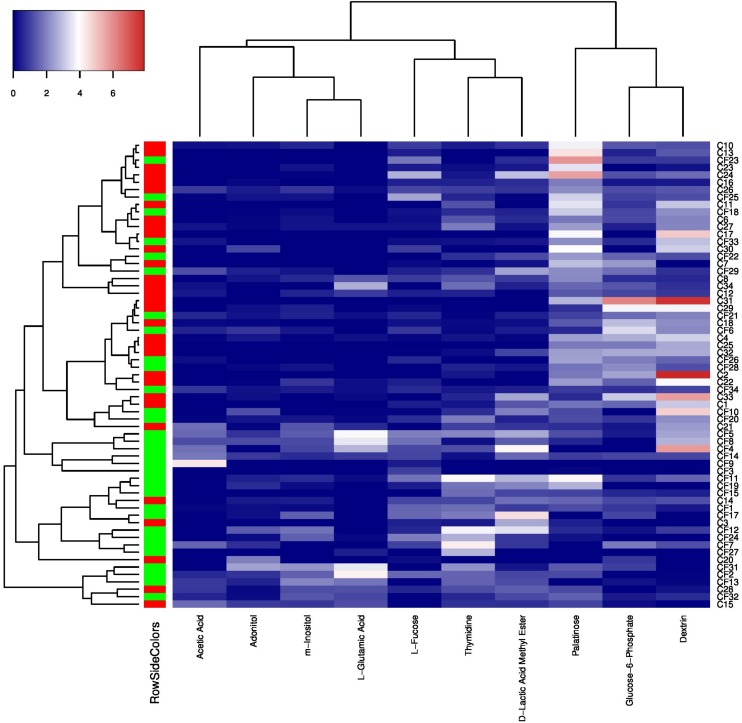
Heatmap cluster analyses of carbon sources utilization patterns and caries states based on 24 h transformed data. The color key represents transformed data, the green RowSideColor represents CF group and the red RowSideColor represent CA group. The carbon sources’ names were listed at the bottom of the heatmap and the sample numbers were listed at the right of the heatmap.

**FIGURE 5:**
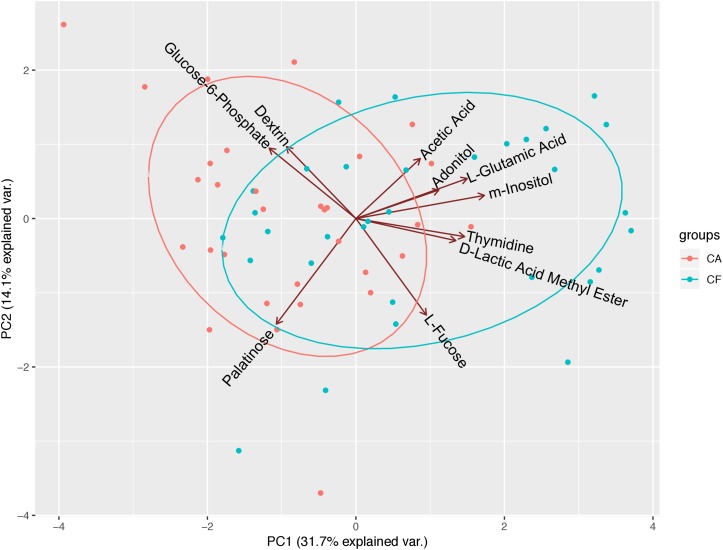
Principal component analyses (PCAs) of microbial community’s utilization patterns in CF and CA groups. The 10 significantly differentially catabolized carbon sources between the CF and CA groups at 24 h were analyzed using transformed data by PCAs. PC1 and PC2 accounted for 31.7 and 14.1% of the total variance. The green dots represent samples from CF group and red dots represent samples from CA group. The green ellipse and red ellipse represent 95% confident interval of CF and CA group, respectively.

To further investigate the correlation between caries states (healthy vs. diseased) and the community-level metabolic functionality of their associated plaque microbiota, heatmap cluster and principal component analyses (PCAs) were performed using the 10 significantly differentially utilized carbon sources between the CF and CA groups identified at 24 h after incubation, a timepoint that can best reflect the community-level utilization rate of specific carbon source (Supplementary Table S3). The results showed that of the 63 subjects (32 from the CF group and 31 from the CA group), majority of them were more closely clustered based on their health states, which was in overall agreement with their clinical diagnosis (Figure 4). Among the 10 carbon sources, palatinose, glucose-6-phosphate, and dextrin are carbohydrates and were classified as a separate cluster with a utilization pattern distinct from the rest of the carbon sources. The second cluster comprised two sub-clusters: one including acetic acid (organic acid), adonitol (carbohydrate), m-inositol (carbohydrate), and L-glutamic acid (amino acid) and the other consisting of L-fucose (carbohydrate), thymidine (nucleoside), and D-lactic acid methyl ester (organic acid). PCAs were performed to further identify the contribution of carbon sources to the different utilization patterns between the CA and CF groups. We included two principle components (PCs) in the analyses. The PCA plot (Figure 5) showed that PC1 and PC2 accounted for 31.7 and 14.1% of the total variance, respectively. The two groups can be readily differentiated by PC1, where plaque microbiota from CA group tend to catabolize dextrin, glucose-6-phosphate, and palatinose more readily than CF group; while acetic acid, adonitol, m-inositol, L-glutamic acid, L-fucose, thymidine, and D-lactic acid methyl ester were more readily utilized by CF group.

## Discussion

Diet and plaque microbiota have been proposed as the key factors contributing to the caries etiology. The high dmft score (11.29 ± 3.49) observed in the CA group correlated with the poor dietary habits (such as high frequency in sweet food, beverage intake and prolonged night feeding). Our data are in agreement with previous studies (Akarslan et al., 2008), and support the “caries tetralogy” concept where “diet” plays a significant role in the etiology of dental caries (Newbrun, 1982).

The acid production as a result of plaque bacterial fermentation of food, particularly cariogenic carbohydrates (such as sweet food and beverage), not only decreases local pH and leads to demineralization of tooth hard tissue, but also further modulates the community toward a more dysbiotic and cariogenic plaque microbiome, augmenting the cycle that promotes cariogenesis. Thus, the plaque microbial activity and especially its community-level metabolic diversity could largely reflect the characteristic physiological features of the plaque microbiota associated with different caries states.

Our Biolog data showed that plaque microbiota associated with CA group has 17 more core-positive (used by plaque microbiome from >90% of subjects within the group) carbon sources, in addition to the 14 shared with CF group (Figure 2). Furthermore, 12 additional non-core carbon sources in category 3 can be found in the CA group, while there is only one carbon source in this category in the CF group. These results suggested that the microbiota of CA children have a broader metabolic spectrum than the microbiota of CF children, indicating a bacterial community with high metabolic versatility and potentially high tolerance to nutrient-limitation.

Interestingly, although the AWCD data revealed a consistently higher overall metabolic activity of plaque microbiota from CA group than CF group at all time points measured, they were not statistically significant (Figure 1). Since AWCD value provides combined information of (1) how many different carbon sources can be utilized; and (2) how extensively certain specific carbon source can be catabolized by the microbial community, it is possible that while plaque microbiota from CA group are capable of utilizing more diverse carbon source (Figure 2), the catabolism might be less effective; on the other hand, although microbiota associated with CF group might utilize less carbon sources, they do so more effectively. Our data are consistent with a recent finding where plaque microbiota associated with severe early childhood caries (SECC) displayed higher metabolic diversity compared to CF control group (Zhao et al., 2017).

Importantly, sucrose, glucose, and raffinose were found to be core-positive carbon sources of the CA group but not of the CF group, and the cariogenic nature of these three carbohydrates have been well documented. Sucrose is of great importance in the initiation of caries due to two factors. First, it can be fermented by bacteria to generate energy, produce acid, and significantly decrease pH; second, it also can function as a substrate, facilitating the synthesis of glucan by plaque bacteria, which helps the bacteria become more adhesive to each other and the dental surface (Colby and Russell, 1997; Thompson et al., 2001). Intraoral cariogenicity tests showed that there was no detectable difference between the effects of glucose and raffinose on cariogenesis compared to sucrose (Koulourides et al., 1976). Furthermore, Nagasawa et al. (2017) found that raffinose and sucrose have cooperative effects, with raffinose inducing biofilm formation by *S*. *mutans* at low sucrose concentration by providing supportive elements that mainly consist of environmental DNA and fructan.

Our data also revealed 7 core-positive carbon sources that can be utilized by 100% of individuals within the CA group. Among which, α-D-lactose, lactulose, arbutin, and salicin were also core-positive (utilized by >90% of the individual) carbon sources of the CF group; while *N*-acetyl-D-glucosamine, palatinose, and D-sorbitol can be utilized by only 81.3, 84.4, and 81.3% of individuals in the CF group (Supplementary Table S2), respectively. Intriguingly, among these 7 carbon sources, only lactose is regarded as cariogenic, which can be utilized by oral bacteria to produce organic acid metabolites and cause demineralization on tooth enamel (Aimutis, 2012). The rest of the carbon sources are generally considered to be non-cariogenic (Ooshima et al., 1983; Touger-Decker and van Loveren, 2003; Gupta, 2018). Our data indicate that not all core-positive carbon sources in the CA group are cariogenic. However, the increased metabolic versatility could enable the plaque microbiota of the CA group to better cope with fluctuating environmental conditions.

The results of utilization of individual carbon source (Figure 3) are largely in agreement with the overall utilization pattern data. Of all the 14 carbon sources that displayed significant differences in utilization (*P* < 0.05) between the CF and CA groups at any of the timepoints (24, 48, 72, and 96 h) following incubation, six of them (including Dextrin, Glucose-6-phosphate, ß-Methyl-D-galactoside, palatinose, pyruvic acid and thymidine-5′-Monophosphate) are better catabolized by CA group and belong to either the core-positive carbon sources of CA group (category 1 and 2 in Figure 2) or non-core carbon source but utilized more frequently by CA group (category 3 of CA group in Figure 2). The rest of the 8 carbon sources seems to be better utilized by CF group. Intriguingly, even though L-Fucose and D-lactic acid methyl ester were more frequently used by CA group (Figure 2), CF group clearly showed higher level of utilization of these two compounds (Figure 3), suggesting that compared to CA group, there were less individual within CF group whose plaque microbial community were able to metabolize these two carbon sources. However, for those capable of utilizing these carbon sources, they did so more effectively than CA group. This may also partly explain why CA group has more core and non-core carbon sources than CF group, but there was no significant difference in AWCD value between the two groups.

The heatmap cluster (Figure 4) and PCAs (Figure 5) of the 10 carbon sources utilized differently between the CF and CA groups both showed that greater utilization of dextrin, glucose-6-phosphate, and palatinose was better correlated with the caries state. Relatively higher utilization of acetic acid, adonitol, m-inositol, L-glutamic acid, L-fucose, thymidine, and D-lactic acid methyl ester may indicate a relatively healthy state. In the heatmap cluster, few subjects from each group were mixed with the other group after clustering. Particularly, a subset of CF subjects was clustered together with CA group based on Biolog analysis. This observation coincides with our previous finding (Xu et al., 2018) which showed that the microbiota could shift prior to the onset of caries, suggesting that the CF subjects classified into the CA group based on Biolog assay could be at risk of developing caries in the near future, or already at the sub-clinical or very early stage of dental caries which might have escaped the clinical examination. Furthermore, Tanner et al. (2011) found that the SECC group can be separated into two subgroups with either a stronger bacterial component or a stronger dietary component using a partial least-squares model, indicating that SECC is diverse, influenced mainly by bacteria or diet. This may partly explain the clustering of five CA subjects together with the CF group based on Biolog analysis, suggesting that even with metabolic capability similar to that of CF associated microbiome, the diets may play a more important role in these subjects in determining their caries outcome.

Compared to microbial community composition, which could shift without significantly impacting community functionality, community level metabolic capability and diversity would be an informative indicator to provide more accurate functional potential of the microbiota and better correlated with healthy and diseased states. Our study clearly revealed higher metabolic versatility displayed by the plaque microbiota associated with CA group compared to CF group, including more universal capability to utilize cariogenic carbohydrates which could contribute to caries development. Our data also provided additional line of evidence to show that, Biolog could be an easy and effective tool in understanding the community-level functional diversity and metabolic versatility of microbiota associated with heathy and diseased states within oral cavity.

## Data Availability Statement

All datasets generated for this study are included in the article/Supplementary Material.

## Ethics Statement

The studies involving human participants were reviewed and approved by The Ethics Committee of the Peking University Health Science Center. Written informed consent to participate in this study was provided by the participants’ legal guardian/next of kin.

## Author Contributions

JT collected the samples, performed the experiments, analyzed the data, and wrote the manuscript. WS analyzed the data. HX designed the experiments and collected the samples. GW collected the samples. XH critically revised the manuscript. FC conceived the work, designed the experiments. MQ conceived the work and designed the experiments, and critically revised the manuscript.

## Conflict of Interest

The authors declare that the research was conducted in the absence of any commercial or financial relationships that could be construed as a potential conflict of interest.
