# Supplementary material for: Differences in Sole Carbon Source Utilization of the Dental Plaque Microbiota Between Caries-Free and Caries-Affected Children
Source: Front Microbiol. 2020 Mar 20;11:458. doi: 10.3389/fmicb.2020.00458 (PMC7100615; doi:10.3389/fmicb.2020.00458)
Supplement: Supplementary file 1 [file Data_Sheet_1.DOCX]

Supplementary Material

# Supplementary Tables

**Table S1. A contrast well without carbon source (A1 well) and 95 sole carbon sources (A2-H12 wells) in the Biolog AN microplate**

| A1  Water | A2  N-Acetyl-D-  Galactosamine | A3  N-Acetyl-D-  Glucosamine | A4  N-Acetyl-β-D-Mannosamine | A5  Adonitol | A6  Amygdalin | A7  D-Arabitol | A8  Arbutin | A9  D-Cellobiose | A10  a-Cyclodextrin | A11  ß-Cyclodextrin | A12  Dextrin |
| --- | --- | --- | --- | --- | --- | --- | --- | --- | --- | --- | --- |
| B1  Dulcitol | B2  i-Erythritol | B3  D-Fructose | B4  L-Fucose | B5  D-Galactose | B6  D-Galacturonic  Acid | B7  Gentiobiose | B8  D-Gluconic Acid | B9  D-Glucosaminic  Acid | B10  a-D-Glucose | B11  Glucose-1-Phosphate | B12  Glucose-6-Phosphate |
| C1  Glycerol | C2  D,L-a-Glycerol  Phosphate | C3  m-Inositol | C4  a-D-Lactose | C5  Lactulose | C6  Maltose | C7  Maltotriose | C8  D-Mannitol | C9  D-Mannose | C10  D-Melezitose | C11  D-Melibiose | C12  3-Melthyl-D-Glucose |
| D1  a-Methyl-D-Galactoside | D2  ß-Methyl-D-Galactoside | D3  a-Methyl-D-  Glucoside | D4  ß-Methyl-D-Glucoside | D5  Palatinose | D6  D-Raffinose | D7  L-Rhamnose | D8  Salicin | D9  D-Sorbitol | D10  Stachyose | D11  Sucrose | D12  D-Trehalose |
| E1  Turanose | E2  Acetic Acid | E3  Formic Acid | E4  Fumaric Acid | E5  Glyoxylic Acid | E6  a-Hydroxybutyric  Acid | E7  ß-Hydroxybutyric  Acid | E8  Itaconic | E9  a-Ketobutyric Acid | E10  a-Ketovaleric Acid | E11  D,L-Lactic Acid | E12  L-Lactic Acid |
| F1  D-Lactic Acid Methyl Ester | F2  D-Malic Acid | F3  L- Malic Acid | F4  Propionic Acid | F5  Pyruvic Acid | F6  Pyruvic Acid  Methyl Ester | F7  D-Saccharic Acid | F8  Succinamic Acid | F9  Succinic Acid | F10  SuccinicAcid  Mono-Methyl Ester | F11  m-Tartaric Acid | F12  Urocanic Acid |
| G1  L-Alaninamide | G2  L-Alanine | G3  L-Alanyl-L-Glutamine | G4  L-Alanyl-L-histidine | G5  L-Alanyl-L-Threonine | G6  L-Asparagine | G7  L-Glutamic Acid | G8  L-Glutamine | G9  Glycyl-L-Aspartic  Acid | G10  Glycyl-L-Glutamine | G11  Glycyl-L-methionine | G12  Glycyl-L-Proline |
| H1  L-Methionine | H2  L-Phenylalanine | H3  L-Serine | H4  L-Threonine | H5  L-Valine | H6  L-Valine plus L-Aspartic Acid | H7  2’-Deoxy | H8  Inosine | H9  Thymidine | H10  Uridine | H11  Thymidine-5’-Monophosphate | H12  Uridine-5’-Monop |

Among the 95 carbon sources, A2-E1 wells belong to carbohydrates; E2-F12 wells belong to organic acids; G1-H6 wells belong to amino acids, peptides and related chemicals; H7-H12 belong to nucleotides and nucleoside.

**Table S2 95 sole carbon sources utilization percentages of the CF and CA children**

| wells | 95 sole carbon sources | percentage of CF individuals (%) | percentage of CA individuals (%) |
| --- | --- | --- | --- |
| B1 | Dulcitol | 62.5 | 67.7 |
| C1 | Glycerol | 46.9 | 61.3 |
| D1 | α-Methyl-D-Galactoside | 78.1 | 77.4 |
| E1 | Turanose | 78.1 | 96.8 |
| F1 | D-Lactic Acid Methyl Ester | 75.0 | 87.1 |
| G1 | L-Alaninamide | 34.4 | 48.4 |
| H1 | L-Methionine | 56.3 | 48.4 |
| A2 | N-Acetyl-D-Galactosamine | 84.4 | 74.2 |
| B2 | i-Erythritol | 62.5 | 54.8 |
| C2 | D,L-α-Glycerol Phosphate | 71.9 | 71.0 |
| D2 | ß-Methyl-D-Galactoside | 78.1 | 87.1 |
| E2 | Acetic Acid | 53.1 | 38.7 |
| F2 | D-Malic Acid | 40.6 | 32.3 |
| G2 | L-Alanine | 46.9 | 51.6 |
| H2 | L-Phenylalanine | 53.1 | 38.7 |
| A3 | N-Acetyl-D-Glucosamine | 81.3 | 100.0 |
| B3 | D-Fructose | 90.6 | 93.5 |
| C3 | m-Inositol | 78.1 | 64.5 |
| D3 | α-Methyl-D-Glucoside | 71.9 | 83.9 |
| E3 | Formic Acid | 68.8 | 61.3 |
| F3 | L-Malic Acid | 40.6 | 45.2 |
| G3 | L-Alanyl-L-Glutamine | 65.6 | 58.1 |
| H3 | L-Serine | 37.5 | 38.7 |
| A4 | N-Acetyl-β-D-Mannosamine | 93.8 | 93.5 |
| B4 | L-Fucose | 78.1 | 83.9 |
| C4 | α-D-Lactose | 90.6 | 100.0 |
| D4 | ß-Methyl-D-Glucoside | 90.6 | 93.5 |
| E4 | Fumaric Acid | 62.5 | 48.4 |
| F4 | Propionic Acid | 37.5 | 32.3 |
| G4 | L-Alanyl-L-histidine | 37.5 | 48.4 |
| H4 | L-Threonine | 31.3 | 19.4 |
| A5 | Adonitol | 56.3 | 51.6 |
| B5 | D-Galactose | 81.3 | 93.5 |
| C5 | Lactulose | 96.9 | 100.0 |
| D5 | Palatinose | 84.4 | 100.0 |
| E5 | Glyoxylic Acid | 31.3 | 22.6 |
| F5 | Pyruvic Acid | 78.1 | 90.3 |
| G5 | L-Alanyl-L-Threonine | 37.5 | 41.9 |
| H5 | L-Valine | 43.8 | 35.5 |
| A6 | Amygdalin | 87.5 | 96.8 |
| B6 | D-Galacturonic Acid | 71.9 | 71.0 |
| C6 | Maltose | 90.6 | 93.5 |
| D6 | D-Raffinose | 87.5 | 96.8 |
| E6 | α-Hydroxy butyric Acid | 68.8 | 87.1 |
| F6 | Pyruvic Acid Methyl Ester | 68.8 | 87.1 |
| G6 | L-Asparagine | 25.0 | 48.4 |
| H6 | L-Valine plus L-Aspartic Acid | 53.1 | 45.2 |
| A7 | D-Arabitol | 62.5 | 61.3 |
| B7 | Gentiobiose | 90.6 | 96.8 |
| C7 | Maltotriose | 93.8 | 93.5 |
| D7 | L-Rhamnose | 90.6 | 90.3 |
| E7 | ß-Hydroxybutyric Acid | 50.0 | 48.4 |
| F7 | D-Saccharic Acid | 43.8 | 29.0 |
| G7 | L-Glutamic Acid | 53.1 | 45.2 |
| H7 | 2’-Deoxy Adenosine | 71.9 | 71.0 |
| A8 | Arbutin | 96.9 | 100.0 |
| B8 | D-Gluconic Acid | 84.4 | 87.1 |
| C8 | D-Mannitol | 81.3 | 93.5 |
| D8 | Salicin | 96.9 | 100.0 |
| E8 | Itaconic | 6.3 | 6.5 |
| F8 | Succinamic Acid | 59.4 | 67.7 |
| G8 | L-Glutamine | 46.9 | 54.8 |
| H8 | Inosine | 71.9 | 87.1 |
| A9 | D-Cellobiose | 90.6 | 96.8 |
| B9 | D-Glucosaminic Acid | 68.8 | 64.5 |
| C9 | D-Mannose | 84.4 | 96.8 |
| D9 | D-Sorbitol | 81.3 | 100.0 |
| E9 | α-Ketobutyric Acid | 46.9 | 45.2 |
| F9 | α-Succinic Acid | 81.3 | 93.5 |
| G9 | Glycyl-L-A spartic Acid | 37.5 | 41.9 |
| H9 | Thymidine | 87.5 | 87.1 |
| A10 | α-Cyclodextrin | 93.8 | 96.8 |
| B10 | α-D-Glucose | 87.5 | 96.8 |
| C10 | D-Melezitose | 90.6 | 93.5 |
| D10 | Stachyose | 78.1 | 96.8 |
| E10 | α-Ketovaleric Acid | 43.8 | 38.7 |
| F10 | Succinic Acid Mono-Methyl Ester | 78.1 | 83.9 |
| G10 | Glycyl-L-Glutamine | 43.8 | 54.8 |
| H10 | Uridine | 71.9 | 74.2 |
| A11 | ß-Cyclodextrin | 78.1 | 90.3 |
| B11 | Glucose-1-Phosphate | 81.3 | 90.3 |
| C11 | D-Melibiose | 84.4 | 90.3 |
| D11 | Sucrose | 81.3 | 93.5 |
| E11 | D,L-Lactic Acid | 59.4 | 74.2 |
| F11 | m-Tartaric Acid | 40.6 | 32.3 |
| G11 | Glycyl-L-methionine | 31.3 | 48.4 |
| H11 | Thymidine-5’-Monophosphate | 56.3 | 80.6 |
| A12 | Dextrin | 53.1 | 80.6 |
| B12 | Glucose-6-Phosphate | 62.5 | 80.6 |
| C12 | 3-Melthyl-D-Glucose | 59.4 | 67.7 |
| D12 | D-Trehalose | 65.6 | 80.6 |
| E12 | L-Lactic Acid | 53.1 | 64.5 |
| F12 | Urocanic Acid | 18.8 | 25.8 |
| G12 | Glycyl-L-Proline | 34.4 | 51.6 |
| H12 | Uridine-5’-Monophosphate | 46.9 | 54.8 |

**Table S3. Carbon sources utilized different between CF and CA group**

| **Type** | **Wells** | **Carbon sources** | **24h** | | **48h** | | **72h** | | **96h** | |
| --- | --- | --- | --- | --- | --- | --- | --- | --- | --- | --- |
|  |  |  | CF | CA | CF | CA | CF | CA | CF | CA |
| Carbohydrates and sugar | A5 | Adonitol | ↑ |  |  |  | ↑ |  |  |  |
|  | A12 | Dextrin |  | ↑ |  |  |  |  |  |  |
|  | B4 | L-Fucose | ↑ |  | ↑ |  | ↑ |  |  |  |
|  | B12 | Glucose-6-Phosphate |  | ↑ |  | ↑ |  | ↑ |  | ↑ |
|  | C3 | m-Inositol | ↑ |  |  |  |  |  |  |  |
|  | D2 | ß-Methyl-D-Galactoside |  |  |  | ↑ |  |  |  |  |
|  | D5 | Palatinose |  | ↑ |  | ↑ |  |  |  |  |
| Organic acids | E2 | Acetic Acid | ↑ |  |  |  |  |  |  |  |
|  | F1 | D-Lactic Acid Methyl Ester | ↑ |  |  |  |  |  |  |  |
|  | F6 | Pyruvic Acid Methyl Ester |  |  |  | ↑ |  | ↑ |  | ↑ |
| Amino acids, peptides and related chemicals | G7 | L-Glutamic Acid | ↑ |  |  |  |  |  |  |  |
|  | H2 | L-Phenylalanine |  |  |  |  |  |  | ↑ |  |
| Nucleotides and nucleoside | H9 | Thymidine | ↑ |  |  |  |  |  |  |  |
|  | H11 | Thymidine-5’-Monophosphate |  |  |  |  |  | ↑ |  | ↑ |
|  | **Total** | | **10** | | **5** | | **5** | | **4** | |
